# America and Her Hospital Workers

**Published:** 1891-11-21

**Authors:** 


### The Hospital

A Weekly Journal of

**Science, Medicine, Nursing, and Philanthropy.**

*For Hospitals, Asylums, Infirmaries, Dispensaries, Medical Practitioners, Students, Nurses, and the Charitable Public.*

Vol. XI.—No. 269.] [Nov. 21, 1891.

### America and her Hospital Workers.

It need not surprise any thinking person that the United States of America, as we announced last week, have decided to establish a National Pension Fund for their trained nurses and hospital workers. The names of those who constitute the Committee of Organisation, and which include the Primus of the American Episcopal Church, Bishop Potter, Mr. Pierpoint Morgan, Mr. C. Vanderbilt, Dr. Billings, Dr. Polk, Dr. Hurd, Rev. Dr. Baker, Dr. Samuel Eliot, Mrs. W. P. Rogers, Dr. Cowles, Dr. Putnam, and Mr. Tatlock represent a governing body of financial, actuarial, and philanthropic strength. The United States have an enthusiastic confidence in the destiny of their country as a leader of nations. In missionary, civilising, and similar humanitarian enterprises they are pioneers, or successors of pioneers, in almost every quarter of the globe. It is not surprising, therefore, that when leading American philanthropists and physicians saw what had been done to elevate the character and promote the prosperity and happiness of nurses and other workers among the sick in the mother country, they should have been fired with a generous emulation to render a like worthy service to their own nurses and hospital workers at home.

The thing which the leaders of American thought and action whom we have named have decided to do is not only great and good in itself, but is particularly opportune at the present moment. It establishes a second precedent for the guidance of Mr. Chamberlain, Dr. Hunter, and other public men who are bent upon disestablishing the poor-house, and re-endowing the aged workman's home. The Royal National Pension Fund of this country was a first object lesson in National Insurance. The National Pension Fund for the hospital workers of the United States is a second; and it has the additional advantage of being tried in another country, and under other circumstances, than our own. It is easy to see that these two Pension Funds, which less than ten years ago had only begun to be thought about, and which had their modest origin in the undeserved hardships of one sick nurse who ended her days in the poor-house—it is easy, we say, to see that these may be the beginnings of a world-wide system of State Insurance, which shall in the long run abolish the poor laws, and preserve the freedom and manhood of every deserving workman to the last day of his life.

England and America, having once entered upon a new departure of this kind, are not likely to let the grass grow under their feet. A love of freedom is a constituent element of the blood of the English-speaking peoples. They love freedom for themselves, and they love it for one another; nay, they love it for all the nations of the earth. If the history of the last three centuries shows anything at all, it shows, not only England "pluming herself" for freedom, but claiming for oppressed nationalities and peoples of every class and colour the same imperishable inheritance. We who live at the present day look back upon noble conquests gloriously won. Not always were our forefathers fully conscious of the greatness of the battles they were fighting, or of the immortal nature of the seed they were sowing for all future time. And it may be that some of those who have so enthusiastically put forth their hands to aid in the building up of these National Pension Funds for hospital workers, have not seen with unclouded vision the true nature of the work they were doing. Possibly even the founder himself did not at first look so far ahead. But the germ of a great idea has been well planted, and now it has come to birth. The principle of self-help has become embodied in two vital and concrete national organisations. Those two may be the parents of thousands of similar institutions. It is often said that "God helps those who help themselves," but if it is once universally realised by all classes of earnest and honest but poorly paid workers, that the State, or private associations of statesman like persons, will help those who help themselves, and will help them in proportion to the energy of their own self-help, then it is certain that new life-blood will be sent coursing and vitalising to the remotest extremities of every political organisation in the world.

The conflict between socialism and individualism is but just beginning. It will be fought out to the end. Undoubtedly, a better state of things will follow if the just, and the true, and the courageous men and women of all faiths and parties will stand firmly to the everlasting principle of justice between man and man, and to the Christian and universal obligation of doing as they would be done by. The men who have joined together to carry out, on a national scale, the pensioning of hospital workers have anticipated the demands of the socialists. They have begun to do, on the broadest possible basis, exactly as they would be done by. They have recognised the duty of hospital workers to practise self-denial and self-help, so far as may be within their powers; but they have also recognised that not even hospital workers can accomplish the impossible, and, therefore, they have stepped in to make round and complete what could only be rendered partial and inadequate by self help alone. Will not something of this kind be the final outcome of the conflict between socialism and individualism?

But it is not as object lessons for statesmen only that these two National Pension Funds demand consideration. They train the minds of the industrial classes in the principles and methods of immediate self-help, and of effective forethought for the future. The cultivated and capable leaders of men are apt to forget that, for the purposes of practical life, the great majority of the rank and file are in a condition of grown-up childhood. Their idea is like that of the lion hunting for his prey; when one meal, or one day, or one week is provided for, they are content, and leave the future to take thought for itself. But this is fatal. It must be remembered that the thoughtless many increase with a rapidity out of all proportion to the increase of the thoughtful few. The work of caring for the many in our overgrown populations is rapidly becoming Herculean and impossible. The only way of hope for the future is in teaching the many to take thought for themselves.

The two National Pension Funds now established deal with a class of people which, until lately, threatened to become grievously burdensome to the already overburdened few. They deal with intelligent, well intentioned, and industrious women of the middle class. Now women, it will be generally admitted, exercise, as a rule, less of forethought than men. If, therefore, the English and American Pension Funds should succeed, by the co-operation of hospitals and hospital managers with nurses, in bringing all, or practically all, the nursing world within the shelter of their provident folds, then one important and peculiarly difficult section of the industrial classes will have solved the problem of immediate and life long self help.

The co-operation of hospitals and their managers, already alluded to, is indispensable to the highest success of these national movements. The authorities of the two funds should, undoubtedly, at once charge themselves with the conversion of hospital managers to the principle of co-operation. Possibly the success of the English Royal National Pension Fund has been almost too great up to the present time. The managers have secured the chief objects which three years ago they aimed at in the establishment of the Fund, and they are, perhaps, inclined to think that it is good to leave well alone. But let them ask themselves, with statesmanlike intelligence, whether it is more to be desired that these two Pension Funds should begin and end with hospital nurses and workers only, or should become the parents and pioneers of numbers of National Pension Funds for all classes of the industrial population? Hospitals and their managers have the power, by enthusiastic co-operation, to bring in the whole of the hospital world in England, her colonies, and the United States. Convert the hospital managers to co-operation, and you not only secure the permanency of the Funds for hospital workers, but you establish two parent organisations whose offspring may in the fulness of time spread over the whole earth.

This is not the place for eulogy either of the two National Pension Funds or their founder; but the writer, as a deeply-interested spectator and friend of the movement, may be permitted to express the conviction that the principles on which the Funds are founded, and the substantial and enduring advantages they are capable of conferring upon their members, entitle them to the deepest sympathy and the prompt co operation of all persons who are anxious to render lasting service to every class of their fellow-creatures, both men and women.

